# Nightshift work can induce oxidative DNA damage: a pilot study

**DOI:** 10.1186/s12889-023-15742-4

**Published:** 2023-05-15

**Authors:** Yutong Zou, Xiaoli Ma, Qian Chen, Ermu Xu, Jialei Yu, Yueming Tang, Danchen Wang, Songlin Yu, Ling Qiu

**Affiliations:** 1 Department of Laboratory Medicine, Peking Union Medical College, Peking Union Medical College Hospital, Chinese Academy of Medical Sciences, No. 1 Shuaifu Yuan, Dongcheng District, Beijing, 100730 PR China; 2 Medical Science Research Center (MRC), Peking Union Medical College, Peking Union Medical College Hospital, Chinese Academy of Medical Sciences, Beijing, 100730 China; 3 State Key Laboratory of Complex Severe and Rare Diseases, Peking Union Medical College, Peking Union Medical College Hospital, Chinese Academy of Medical Sciences, No. 1 Shuaifu Yuan, Dongcheng District, Beijing, 100730 PR China

**Keywords:** Night shift, Nucleic-acid damage, 8-oxo-7,8-dihydroguanosine, 8-oxo-7,8-dihydro-2'-deoxyguanosine

## Abstract

**Background:**

Regular sleep is very important for human health; however, the short-term and long-term effects of nightshift with sleep deprivation and disturbance on human metabolism, such as oxidative stress, have not been effectively evaluated based on a realistic cohort. We conducted the first long-term follow-up cohort study to evaluate the effect of nightshift work on DNA damage.

**Methods:**

We recruited 16 healthy volunteers (aged 33 ± 5 years) working night shifts at the Department of Laboratory Medicine at a local hospital. Their matched serum and urine samples were collected at four time points: before, during (twice), and after the nightshift period. The levels of 8-oxo-7,8-dihydroguanosine (8-oxoG) and 8-oxo-7,8-dihydro-2’-deoxyguanosine (8-oxodG), two important nucleic-acid damage markers, were accurately determined based on a robust self-established LC‒MS/MS method. The Mann–Whitney U or Kruskal–Wallis test was used for comparisons, and Pearson’s or Spearman’s correlation analysis was used to calculate the correlation coefficients.

**Results:**

The levels of serum 8-oxodG, estimated glomerular filtration rate-corrected serum 8-oxodG, and the serum-to-urine 8-oxodG ratio significantly increased during the nightshift period. These levels were significantly higher than pre-nightshift work level even after 1 month of discontinuation, but no such significant change was found for 8-oxoG. Moreover, 8-oxoG and 8-oxodG levels were significantly positively associated with many routine biomarkers, such as total bilirubin and urea levels, and significantly negatively associated with serum lipids, such as total cholesterol levels.

**Conclusion:**

The results of our cohort study suggested that working night shifts may increase oxidative DNA damage even after a month of discontinuing nightshift work. Further studies with large-scale cohorts, different nightshift modes, and longer follow-up times are needed to clarify the short- and long-term effects of night shifts on DNA damage and find effective solutions to combat the negative effects.

**Supplementary Information:**

The online version contains supplementary material available at 10.1186/s12889-023-15742-4.

## Introduction

Sleep is an essential physiological state and plays a critical role in various bodily functions, including defense against oxidative stress [1]. However, 18 to 25% of the workforce in industrialized countries works alternate-shift schedules, and this situation is especially prevalent in critical services and public utilities such as healthcare [2]. Nightshift work, along with some extent of sleep deprivation and disturbance in circadian rhythm, was found to be closely associated with adverse health effects such as diabetes, cardiovascular diseases, cancer, and even all-cause mortality [3–5]. It was reported that shift work could alter the pro/antioxidant balance in favor of oxidative stress, inducing functional disorders [4]. In another study, an almost twofold temporary increase in the oxidative stress index was reported after 16 h of continuous shift work, along with an increase in oxidant status and a decrease in antioxidant status [6]. However, previous studies investigated the relationship between sleep disturbance/night shift and oxidative stress by recruiting and interfering volunteers [7], which may not be representatively applicable for a realistic assessment of nightshift workers, or by conducting cross-sectional studies comparing individuals on the night shift with those who are not [8–12]. Even when a follow-up cohort was attempted [13], only temporary changes before and after one round of nightshift work were compared. However, it was also reported that prolonged wakefulness or sleep deprivation can also activate adaptive stress pathways, such as the unfolded protein response, which can guard against the deleterious consequences of oxidative stress [14]. Thus, a follow-up cohort study is still lacking and needed to realistically explore both the short-term and long-term effects of realistic nightshift work on human oxidative stress.

Nucleic-acid damage is more stable than changes in proteins and lipids, and a damaged genome can affect the entire cellular integrity; hence, previous studies are mainly focused on nucleic-acid damage, the most dangerous of all modifications observed among biomolecules, to evaluate oxidative stress in humans [15]. Initiated by DNA and RNA turnover and repair, 8-oxo-7,8-dihydroguanosine (8-oxoG) and 8-oxo-7,8-dihydro-2’-deoxyguanosine (8-oxodG) are extensively considered sensitive and crucial biomarkers of oxidative damage, which also show diurnal rhythms [16, 17]. Since 8-oxoG and 8-oxodG can cross the cell membrane, they can be detected in human urine and/or serum samples, making them effective biomarkers for large-scale applications [18, 19]. Several previous studies have reported significantly higher levels of 8-oxoG and 8-oxodG in brain tissues of mice/rats with sleep disturbance, but no such studies on human beings have been reported thus far [20, 21]. In our laboratory, we have established and verified a robust LC‒MS/MS method for the accurate determination of 8-oxoG and 8-oxodG levels in serum and urine samples [22], which is still lacking in previous reports.

In this pilot cohort study, we recruited volunteers in the “1-1-3” nightshift mode from our clinical laboratory and evaluated whether working night shifts can cause negative effects on oxidative damage to nucleic acids and whether leaving the night shift for 1 month could effectively mitigate those effects.

## Materials and methods

### Chemicals and reagents

Powders of 8-oxoG, [^13^ C, ^15^N_2_] 8-oxoG, 8-oxodG, and [^13^ C, ^15^N_2_] 8-oxodG were purchased from Toronto Research Chemicals (Toronto, Canada). High-performance liquid chromatography-grade methanol, acetonitrile, and formic acid were purchased from Thermo Fisher Scientific (Fair Lawn, NJ, USA). Deionized water was purchased from A.S. Watson Group (Hong Kong, China).

### Participant inclusion and sample collection

**Fig. 1:**
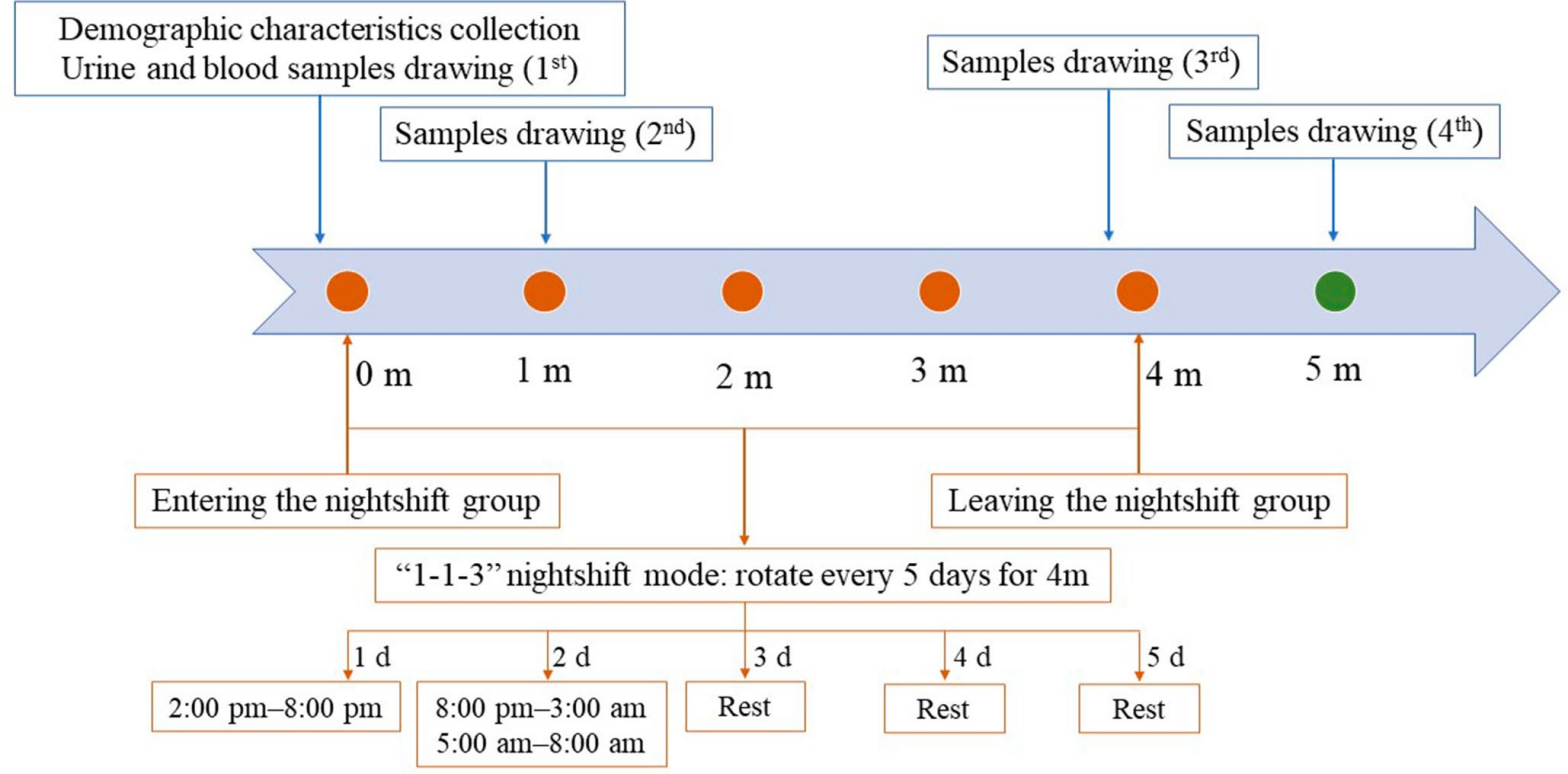
The flowchart of participant inclusion and sample collection

A total of 16 apparently healthy volunteers who worked one-round night shift from January to December 2021 in the Department of Laboratory Medicine of a local hospital were recruited (Fig. 1). Each round of nightshift work lasted 4 months and was rotated every 5 days. The nightshift mode was called the “1-1-3” mode, and it was consisted of a short nightshift (2:00 pm–8:00 pm) on the first day, followed by a long nightshift (8:00 pm–3:00 am and 5:00 am–8:00 am, with a 2-h break between 3:00 am and 5:00 am) on the next day, and then rest for three days. The urine and blood samples were collected at four time points: before entering the nightshift group, 1 month after entering the nightshift group, before leaving the nightshift group (i.e., 4 months after entering the nightshift group) and 1 month after leaving the nightshift group. All serum and urine samples were collected between 8:00 and 9:00 am (the second and third samples were collected after the long nightshift ended) to reduce the possibility of confounding factors. All volunteers were required to maintain a normal diet and abstain from smoking, drinking alcohol, and consuming coffee, tea, or other stimulant food. They were also to avoid strenuous exercise within 24 h of sample collection. After sitting for 10–15 min, fasting blood samples were drawn into red-capped procoagulant-containing 5-mL Vacuette tubes with gel (Greiner Bio-One, Kremsmünster, Austria) and centrifuged at 3000 *rpm* for 10 min at 4°C; random spot urine samples were collected and stored in white-capped 5-mL Vacuette tubes without any additives; the menstrual period of all the female participants was avoided when the urine samples were collected. All serum and urine samples were repacked and stored at -80 °C until uniform measurements.

### Sample preparation and measurement

Serum biochemical measurements, including alanine aminotransferase (ALT), albumin (Alb), total cholesterol (TC), triglycerides (TG), glucose, and urea levels, were analyzed using an AU 5800 Automatic Biochemical Analyzer (Beckman Coulter, Brea, CA, USA), and urine biochemical measurements, including creatinine (Cr) levels, were measured using an Beckman AU 2700 Automatic Biochemical Analyzer.

The levels of 8-oxoG and 8-oxodG in serum and urine samples were measured using the ACQUITY I-Class UPLC system and the TQ-XS triple quadrupole MS/MS system (Waters Corporation, MA, USA) coupled with an ACQUITY™ Primer HSS T3 column (Waters Corporation; 2.1 × 100 mm, 1.8 μm VanGuard™ FIT, Part No. 186009471). The specific method developed by our research group has been previously validated and published [22, 23], and the serum sample preparation process is shown as Supplemental Fig. 1. The measurements of 8-oxoG and 8-oxodG in random spot urine samples were adjusted by dividing by urinary Cr (mmol/L). Since the concentrations of 8-oxoG and 8-oxodG in serum/plasma are mainly dependent on the renal glomerular filtration rate (GFR), the serum levels of 8-oxoG and 8-oxodG were corrected using the estimated GFR (eGFR) [24], which is routinely calculated from equations using serum/plasma Cr, sex, and age. Moreover, the ratio of plasma and urinary 8-oxoG was found to be a novel evaluation index for end-stage renal disease [25]; thus, the ratios of serum and Cr-adjusted urinary 8-oxoG and 8-oxodG were also evaluated in this study.

### Statistical analysis

Statistical analysis was performed using Excel 16.0 (Microsoft, Seattle, WA, USA) and SPSS 22.0 (IBM, Chicago, IL, USA). All figures were drawn by GraphPad Prism 7.0 (GraphPad Software, San Diego, CA, USA). All data are presented as the mean ± standard deviation. The Mann–Whitney U or Kruskal–Wallis test was used for comparisons among groups. Pearson’s or Spearman’s correlation analysis was used to calculate the correlation coefficients (r). Notably, r values between 0.40 and 0.69 represented moderate correlations, whereas those between 0.70 and 0.89 represented strong correlations [26]. Two-sided *p* values < 0.05 indicated statistical significance.

## Results

### Demographic characteristics of the individuals enrolled in the study

A total of 16 volunteers (aged 33 ± 5 years), including 11 females (aged 34 ± 4 years), from the nightshift group were included in this study, and their demographic characteristics when they first entered this cohort study are shown in Table 1. Since only the young workforce (< 45-year-old males; <40-year-old females) worked the night shift at the local hospital, their average age was only 33 years. The distribution of Alb was significantly higher in males (*p* = 0.027) than in females, whereas that of TC was significantly lower in males (*p* = 0.019). Except that the distribution of serum 8-oxodG was significantly higher in females than that in males (*p* = 0.027), no significant difference between males and females was found for the distribution of serum 8-oxoG (*p* = 0.913), urinary 8-oxoG/Cr (*p* = 0.377) and urinary 8-oxodG/Cr (*p* = 0.441).

**Table 1:** The demographic characteristics of the 16 volunteers

	Male (n = 5)	Female (n = 11)	Total (n = 16)
Age (year)	31 ± 7	34 ± 4	33 ± 5
BMI (kg/m^2^)	22.1 ± 4.1	22.4 ± 3.4	22.3 ± 3.5
SBP (mmHg)	113.4 ± 3.1	110.9 ± 12.5	111.7 ± 10.4
ALT (U/L)	46.8 ± 42.0	19.9 ± 13.1	28.3 ± 27.4
Alb (g/L)	47.2 ± 0.8	45.5 ± 2.4	46.1 ± 2.1
TBil (µmol/L)	14.0 ± 5.5	9.7 ± 3.4	11.1 ± 4.4
Urea (mg/dL)	4.6 ± 1.3	3.9 ± 0.7	4.1 ± 0.9
Glucose (mmol/L)	5.4 ± 0.4	5.4 ± 0.5	5.4 ± 0.4
TC (mmol/L)	3.6 ± 0.4	4.5 ± 0.9	4.2 ± 0.9
TG (mmol/L)	0.6 ± 0.2	1.2 ± 0.7	1.0 ± 0.6
Urinary Cr (mol/mL)	14.1 ± 1.7	13.4 ± 7.4	13.6 ± 6.1
Serum 8-oxoG (ng/mL)	0.048 ± 0.011	0.033 ± 0.008	0.038 ± 0.011
Serum 8-oxodG (ng/mL)	0.019 ± 0.005	0.024 ± 0.013	0.022 ± 0.011
Urinary 8-oxoG/Cr (mg/mol)	0.777 ± 0.120	0.696 ± 0.132	0.721 ± 0.130
Urinary 8-oxodG/Cr (mg/mol)	0.624 ± 0.147	0.698 ± 0.223	0.675 ± 0.200

BMI, body mass index; SBP, systolic blood pressure; ALT, alanine aminotransferase; Alb, albumin; TBil, total bilirubin; TC, total cholesterol; TG, triglycerides; Cr, creatinine. All data are presented as the mean ± standard deviation

### Levels of 8-oxoG and 8-oxodG in serum during the nightshift period

**Fig. 2:**
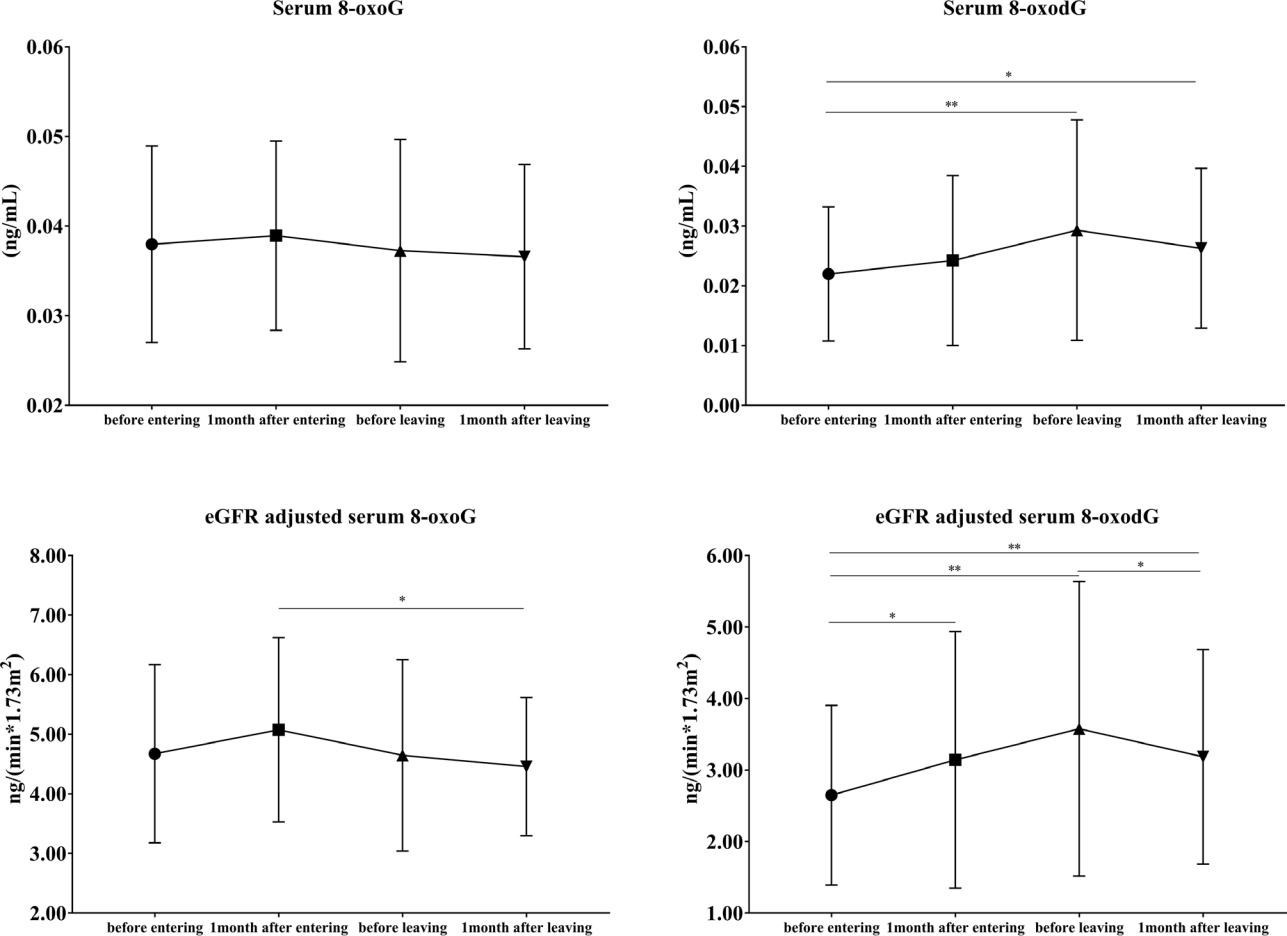
The distribution of serum 8-oxoG and 8-oxodG in 16 volunteers 8-oxoG, 8-oxo-7,8-dihydroguanosine; 8-oxodG, 8-oxo-7,8-dihydro-2’-deoxyguanosine; eGFR, estimated glomerular filtration rate All data are presented as the mean ± standard deviation ^*^p < 0.05, ^**^p ≤ 0.01

As shown in Fig. 2, no significant change was observed in serum 8-oxoG levels during and after the nightshift period. However, serum 8-oxodG was observed to increase gradually during the nightshift period and was found to be significantly higher than the pre-nightshift work level (*p* = 0.009) after 4 months of entering the nightshift round and still significantly higher than the pre-nightshift work level even after 1 month of leaving the nightshift round (*p* = 0.013). The corrected 8-oxoG levels were determined to be significantly lower after 1 month of leaving the nightshift round than after 1 month of entering the nightshift round (*p* = 0.048). The corrected 8-oxodG levels were also significantly higher at 1 and 4 months after entering the nightshift round compared to the pre-nightshift work levels (*p* = 0.040, *p* = 0.005). It decreased significantly after leaving the nightshift round (*p* = 0.030); however, it was still significantly higher than the pre-nightshift work levels (*p* = 0.008).

### Levels of 8-oxoG and 8-oxodG in urine during the nightshift period

**Fig. 3:**
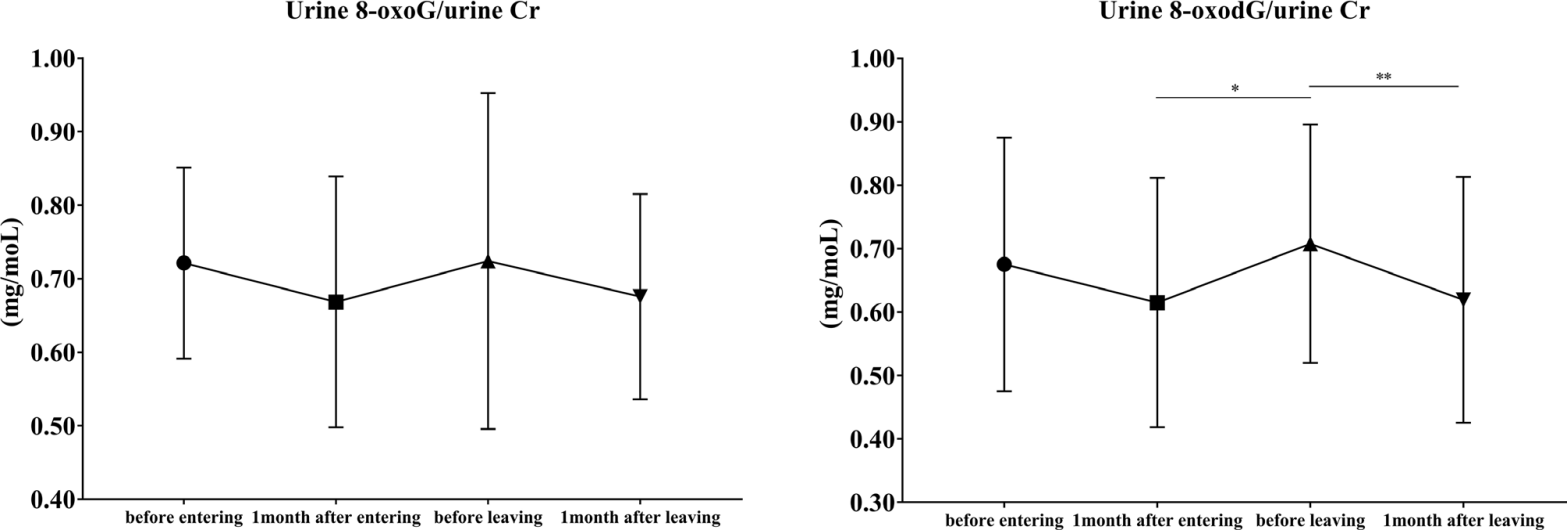
The distribution of urinary 8-oxoG and 8-oxodG in 16 volunteers 8-oxoG, 8-oxo-7,8-dihydroguanosine; 8-oxodG, 8-oxo-7,8-dihydro-2’-deoxyguanosine; Cr, creatine All data are presented as the mean ± standard deviation ^*^p < 0.05, ^**^p ≤ 0.01

Similar to the results for serum 8-oxoG, urinary 8-oxoG/urinary Cr levels did not show a significant change (Fig. 3). However, urinary 8-oxodG/urinary Cr levels were significantly higher after 4 months of entering the nightshift round than after 1 month of entering it (*p* = 0.018); they also showed a significant decrease after 1 month of leaving the nightshift round (*p* = 0.007).

### 8-oxoG and 8-oxodG serum-to-urine ratios during the nightshift period

**Fig. 4:**
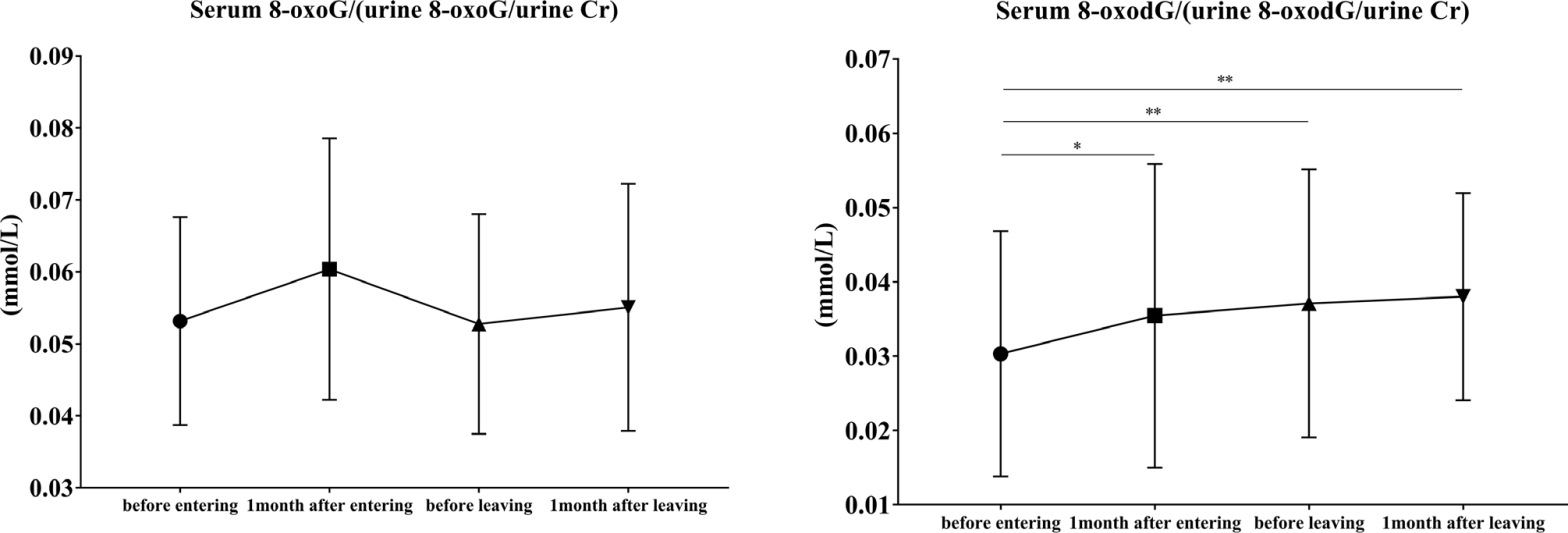
The ratio of 8-oxoG and 8-oxodG in serum and urine in 16 volunteers 8-oxoG, 8-oxo-7,8-dihydroguanosine; 8-oxodG, 8-oxo-7,8-dihydro-2’-deoxyguanosine; Cr, creatine All data are presented as the mean ± standard deviation ^*^p < 0.05, ^**^p ≤ 0.01

Similar to serum and urinary 8-oxoG levels, there was no significant change in the 8-oxoG serum-to-urine ratio (Fig. 4). However, the 8-oxodG serum-to-urine ratio showed a significant increase during the nightshift period (*p* = 0.035, *p* = 0.010) and was found to be significantly higher than the pre-nightshift work level (*p* = 0.006) even after 1 month of the nightshift period.

### Correlation among 8-oxoG, 8-oxodG, and routine biomarkers in serum and urine

The correlation between urinary 8-oxoG and urinary 8-oxodG (*r* = 0.876) was deemed strong, whereas those between urinary 8-oxoG/urinary Cr and urinary 8-oxodG/urinary Cr (*r* = 0.504) and serum 8-oxoG and urinary 8-oxoG/urinary Cr (*r* = 0.469) were deemed to be moderate. Moreover, the correlations between 8-oxoG, 8-oxodG and the routine biomarkers in serum and urine samples were analyzed and are shown in Table 2. Significantly positive correlation between serum 8-oxoG and ALT, total bilirubin (TBil), direct bilirubin, urea, Cr and glucose, and significantly negative correlation between serum 8-oxoG and TC, high density lipoprotein cholesterol (HDL-C) and low density lipoprotein cholesterol (LDL-C) were recognized, however, all these correlations were deemed weak with r < 0.4. Moreover, the weak positive correlations between serum 8-oxodG and TBil and urea, and weak negative correlations between serum 8-oxoG and TC and LDL-C were also found. And only the correlation between serum 8-oxoG and prealbumin was deemed moderate (r = 0.449). The correlations between urinary 8-oxoG and 8-oxodG with many routine urinary biomarkers, including urinary protein, microalbumin, urea, uric acid, potassium and phosphorus, were deemed moderate and even strong. Since the urinary 8-oxoG and 8-oxodG concentrations were strongly correlated with the level of urinary creatinine (r = 0.871, r = 0.799), the correlations between them and the other routine urinary biomarkers were closely related to the urine volume.

**Table 2:** The correlation between 8-oxoG, 8-oxodG and routine biomarkers in serum and urine

	Alb	PA	ALT	TBil	DBil	Urea	Cr	Glucose	TC	TG	HDL-C	LDL-C
**Serum 8-oxoG**	0.076	0.449^**^	0.347^**^	0.332^**^	0.375^**^	0.374^**^	0.355^**^	0.335^**^	-0.384^**^	0.148	-0.366^**^	-0.310^*^
**Serum 8-oxodG**	-0.187	0.242	-0.056	0.272^*^	0.168	0.348^**^	0.141	0.153	-0.299^*^	0.105	-0.016	-0.281^*^
	**U-protein**	**U-MAlb**	**U-Urea**	**U-UA**	**U-Cr**	**ACR**	**PCR**	**U- potassium**	**U-calcium**		**U-phosphorus**	
**Urinary 8-oxoG**	0.761^**^	0.617^**^	0.596^**^	0.631^**^	0.871^**^	0.006	-0.263^*^	0.518^**^	0.253^*^		0.678^**^	
**Urinary 8-oxodG**	0.741^**^	0.661^**^	0.579^**^	0.604^**^	0.799^**^	0.145	-0.211	0.460^**^	0.419^**^		0.670^**^	

Albumin, Alb; PA, prealbumin; ALT, alanine aminotransferase; TBil, total bilirubin; DBil, direct bilirubin; Cr, creatinine; TC, total cholesterol; TG, triglyceride; HDL-C, high density lipoprotein cholesterol; LDL-C, low density lipoprotein cholesterol; U, urinary; MAlb, microalbumin; UA, uric acid; ACR, microalbumin creatinine ratio; PCR, total protein creatinine ratio

^*^p < 0.05, ^**^p < 0.01

## Discussion

In this cohort study, we observed that the “1-1-3” nightshift mode had a significant effect on the levels of 8-oxodG in both serum and urine samples, with and without eGFR correction, which implies that nightshift work could induce oxidative DNA damage. Moreover, the increase in oxidative DNA damage could not be effectively mitigated even after 1 month of normal shift work. Previous studies [16, 17] have reported that oxidative stress markers, including 8-oxodG, show a gradual decrease at night (10:00 pm to 6:00 am) and a gradual increase during the day (7:00 am to 6:00 pm). Thus, sleep time is important for the mitigation of oxidative damage, and working the night shift with circadian disruption may worsen this damage.

However, no significant change was found for 8-oxoG. RNA is more prone to guanine oxidation than DNA because of its single-stranded structure, lack of protective proteins, and cytosolic location closer to the mitochondria [27, 28]. However, the half-life of mRNAs varies considerably from minutes to days, with a median of ~ 12 h [29]. Thus, the three-days’ rest of the “1-1-3” nightshift mode could provide enough time to metabolize the guanine oxidation in the RNA but not enough to metabolize the DNA guanine oxidation, enabling continuous accumulation of 8-oxodG in the human body. It is noteworthy that since the samples at 1 month and 4 months after nightshift work were collected after a long nightshift, their levels reflect not only long-term effects but also acute effects due to the lack of sleep and disruption of circadian rhythms. The fourth sample, however, reflects only long-term effects. Since no significant increase was observed for 8-oxoG levels in the second and third samples in this study, we speculate that the long-term effects are more notable. However, further exploration and assessment of the short and long-term effects of nightshift work on human nucleic-acid damage are still necessary.

Since 8-oxoG and 8-oxodG are released from the cells into the blood stream and excreted through urine by the kidneys, 8-oxoG and 8-oxodG in 24-hour urine samples show a strong correlation with intracellular 8-oxoG and 8-oxodG levels and could represent overall systemic oxidative stress on nucleic acids [27, 30]. It was reported that the levels of 8-oxoG and 8-oxodG, corrected by Cr in random spot urine samples, could replace the results of 24-hour urine samples [31]. Owing to the close association between the levels of 8-oxoG and 8-oxodG in plasma and kidney function, some studies did not consider them as valid measurements of nucleic-acid damage [27]. However, good correlations between urine and plasma concentrations of 8-oxoG and 8-oxodG were also observed, implying that plasma/serum was a suitable matrix in addition to urine [32]. Previous studies have established and applied a physiological model with eGFR to correct serum/plasma measurements [19, 24, 33], which was considered an equally potential measure of oxidative stress on nucleic acids and was therefore also used in this study. Moreover, the ratios of plasma and urinary 8-oxoG are considered a novel evaluation index for end-stage renal disease [25] and were also evaluated. Here, we found that serum 8-oxodG, eGFR-corrected serum 8-oxodG, and the 8-oxodG serum-to-urine ratio could be potential biomarkers for the evaluation of oxidative stress on nucleic acids in humans.

Many previous studies have explored the short- and long-term effects of nightshift work on human oxidative stress, including oxidative damage to nucleic acids. The short-term effects of nightshift work were mainly evaluated by recruiting volunteers and implementing sleep deprivation with/without a sleep recovery intervention. It was found that [34] one day of sleep deprivation could increase human oxidative damage with significantly elevated levels of catalase and malondialdehyde and decreased levels of the antioxidant glutathione and decreased activity of the antioxidant superoxide dismutase. Moreover, the effect of just one day of sleep deprivation on redox metabolites was profound, and it could not be effectively restored after one day of sleep recovery [34]. The long-term effects of night shifts were mainly evaluated based on cross-sectional studies by including different volunteers in day shifts or night shifts. It was reported that nightshift workers had higher levels of oxidative damage and lower levels of antioxidant defenses than those who worked the day shift [8]. Increased oxidative DNA damage and biological aging levels were also found in nightshift workers in another study with higher urinary levels of 8-oxo-7,8-dihydroguanine and shorter leukocyte telomere length [9]. Moreover, the excretion of 8-oxodG among nightshift workers was found to be reduced during daytime sleep, which could reflect reduced functioning of the DNA repair machinery [35]. A systematic review [36] based on 12 correlational studies published in 2001–2019 also found that nightshift work was associated with increased DNA damage, reduced DNA repair capacity, higher levels of reactive oxygen species, and a reduction in antioxidant defense. However, this review also pointed out that the conclusion was limited due to the lack of longitudinal or experimental studies; thus, we conducted a long-term follow-up cohort study to further explore the effects of nightshift work on human health based on the realistic “1-1-3” nightshift mode.

We also observed a significant correlation among 8-oxoG, 8-oxodG, and some routine biomarkers in both serum and urine samples, which is reasonable considering the close correlation between oxidative damage to nucleic acids and these routine biomarkers related diseases, including liver injury, chronic kidney disease, and type 2 diabetes [37–39]. However, studies exploring the relationship and mechanism among 8-oxoG, 8-oxodG, and routine biomarkers are still lacking. Moreover, significantly negative relationships between the levels of 8-oxoG and 8-oxodG in serum and urine samples and that of serum lipids, including TC, HDL-C, and LDL-C, were observed in this study. Further analysis showed that lipid levels decreased during the nightshift period and increased when returning to the normal work schedule, more significantly for TG, which are easily affected by diet. A simple questionnaire revealed that only 3 of these 16 volunteers (male, n = 1) ate snacks to replenish energy during the long nightshift time before their samples were collected. Thus, this phenomenon of changes in lipid levels may be closely related to excessive consumption and insufficient supplementation during the nightshift period. Moreover, a prolonged daily eating duration was likely to occur, which is associated with a higher risk of obesity, diabetes, cardiovascular disease, and so on [40]. Thus, limiting daily eating episodes to a 10–12 h window (or keeping between 6 and 10-h per day, if possible) could be beneficial for the improvement of metabolic health [40, 41]. Other interventions, such as caffeine naps, exogenous melatonin administration, an additional 2-h of sleep in the afternoon before a shift, and 20–45 min naps during the night shift, were suggested to improve vigilance, attention, and subjective fatigue; attenuate circadian misalignment; and improve recovery from nightshift work [42–44].

There are some limitations of this study. Compared to studies on sleep interventions in volunteers, more confounding factors could exist in our cohort study, such as uncontrollable sleep time and eating habits. However, the results of this study are more likely to reflect the real situation. The sample size was small, and only one “1-1-3” nightshift mode was evaluated in this study. Furthermore, the 1-month follow-up time may not enough to offset the observed increase in nucleic-acid damage. Thus, large-scale cohort studies with different nightshift modes and longer follow-up times are needed in the future to comprehend the effect of nightshift work and find effective solutions. Moreover, measuring the yield of 8-oxoguanine DNA N-glycosylase-sensitive sites (mostly 8-oxodG) in the tissues or DNA of leukocytes could be more reliable than serum and urine samples [45], but they were unavailable for this study. Based on the availability of an accurate LC‒MS/MS method, only the levels of 8-oxodG were used to assess the DNA damage status in this study. Even if 8-oxodG is extensively considered as a sensitive and crucial oxidative DNA damage biomarker, some other biomarkers, such as 5-OH uracil, thymine glycol and hydantoins, could be further included to more comprehensively assess human DNA damage status. Thus, although this study addresses a preliminary understanding of the effect of night shifts on oxidative stress, further explorations are still needed.

This study has some strengths as well. To the best of our knowledge, no cohort study has explored the effect of night shifts on the levels of 8-oxoG and 8-oxodG in human body fluids in clinical practice. First, all volunteers were recruited from the Department of Laboratory Medicine of a local hospital and worked the “1-1-3” nightshift mode. The collection time was set at the morning (8:00–9:00 am), after the end of the “long nightshift” to avoid possible confounding factors, as emphasized by a previous study [4]. To our knowledge, this is the first cohort study to explore the effect of night shifts on the damage caused to nucleic acids by oxidative stress in humans using its crucial biomarkers, and the study results indicate that nightshift work induces oxidative DNA damage. Furthermore, the levels of both 8-oxoG and 8-oxodG in the matched serum and urine samples were measured based on our self-established LC‒MS/MS method, which has been comprehensively validated and deemed excellent [22, 23].

To summarize, we established a long-term cohort study to explore the effect of the realistic “1-1-3” nightshift mode on oxidative damage to human nucleic acids. Based on the measurements of both serum and urinary 8-oxodG, the oxidative DNA damage was found to be significantly elevated during the nightshift period, and the effect could not be effectively offset even 1 month after returning to the day shift. Further studies with large-scale cohorts, different nightshift modes, and longer follow-up times are needed to clarify the short- and long-term effects of night shifts on DNA damage, and it is also important to find effective solutions to combat the negative effects.

## Electronic supplementary material

Below is the link to the electronic supplementary material.

Supplementary Material 1

## Data Availability

The datasets used and/or analyzed during the current study are available from the corresponding author on reasonable request.
