# Supplementary material for: Nightshift work can induce oxidative DNA damage: a pilot study
Source: BMC Public Health. 2023 May 15;23:891. doi: 10.1186/s12889-023-15742-4 (PMC10184341; doi:10.1186/s12889-023-15742-4)
Supplement: Supplementary file 1 — Supplementary Material 1 [file 12889_2023_15742_MOESM1_ESM.docx]

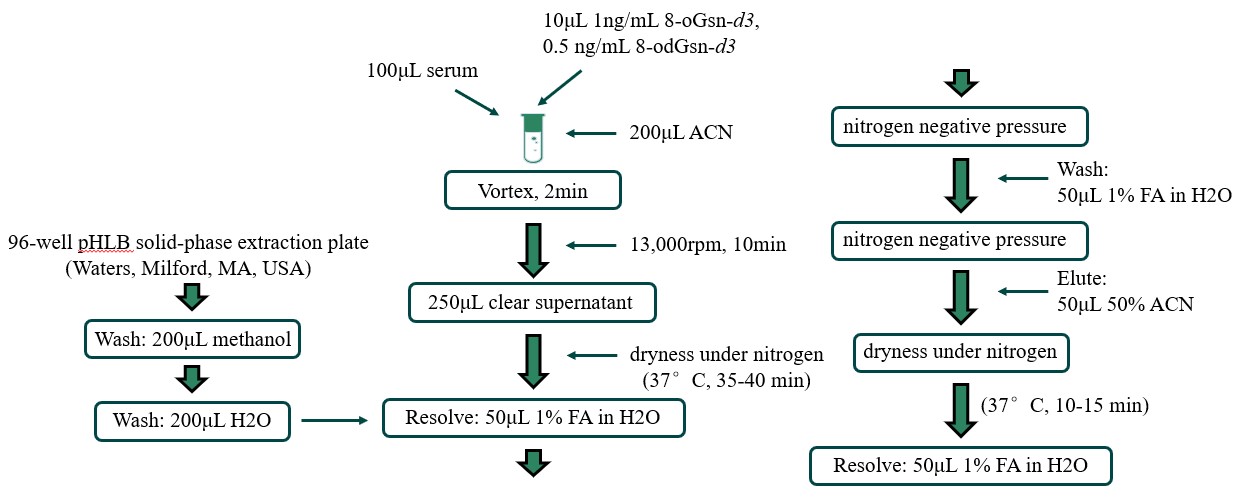


**Supplemental Figure 1 The preparation flow of serum sample**

8-oxoG, 8-oxo-7,8-dihydroguanosine; 8-oxodG, 8-oxo-7,8-dihydro-2'-deoxyguanosine; FA, formic acid; ACN, acetonitrile
